# Phytochemical Profiling, Antioxidant, Antimicrobial and Cytotoxic Activities of *Stipagrostis plumosa* (*Poaceae*): Potential Implications for Human and Veterinary Health

**DOI:** 10.3390/biology15161428

**Published:** 2026-08-19

**Authors:** Rehab M. A. El-Desoukey, Mashail N. AlZain, Fawziah M. Albarakaty

**Affiliations:** 1 Microbiology and Immunology Department, National Research Centre, Giza 12622, Egypt; 2 Department of Biology, College of Sciences, Princess Nourah Bint Abdulrahman University, Riyadh 11451, Saudi Arabia; mnalzain@pnu.edu.sa; 3 Department of Biology, Faculty of Science, Umm Al-Qura University, P.O. Box 715, Makkah 21955, Saudi Arabia

**Keywords:** GC–MS analysis, secondary metabolites, phenolic compounds, flavonoids, natural bioactive compounds, medicinal grasses

## Abstract

*Stipagrostis plumosa* is a desert grass that remains relatively underexplored with respect to its phytochemical composition and biological activities, particularly against pathogens of veterinary relevance. This study aimed to characterize the phytochemical profile of *S. plumosa* and evaluate the antioxidant, antimicrobial, and cytotoxic activities of its extracts and solvent fractions. Aerial parts of *S. plumosa* were collected during the flowering season from Al-Quwai’iyah, Saudi Arabia, and subjected to methanolic extraction followed by solvent fractionation. Total phenolic and flavonoid contents, antioxidant activity, antimicrobial activity against bacterial and fungal strains, and cytotoxicity against MCF-7 and A549 cells were evaluated. GC–MS was used to characterize the ethyl acetate and chloroform fractions. The crude methanolic extract contained 72.1 mg GAE/g extract of total phenolics and 13.6 mg QE/g extract of total flavonoids. The ethyl acetate fraction exhibited the strongest antioxidant activity (IC_50_ = 72.48 µg/mL) and the most pronounced antimicrobial activity, whereas the chloroform fraction showed the highest cytotoxic activity against MCF-7 and A549 cells, with IC_50_ values of 173.52 and 194.17 µg/mL, respectively. GC–MS profiling revealed diverse phenolic derivatives, fatty acids and their esters, oxygenated compounds, and phytosterol-related constituents. Stigmasta-3,5-dien-7-one (14.70%) and vanillic acid (7.18%) predominated in the ethyl acetate fraction, while hexadecanoic acid methyl ester (5.28%) was among the major constituents of the chloroform fraction. Overall, the fraction-dependent biological activities and diverse phytochemical profiles highlight *S. plumosa* as a promising source of natural bioactive compounds with potential relevance to veterinary health and the development of alternative antimicrobial strategies.

## Simple Summary

*Stipagrostis plumosa* is a desert grass adapted to harsh environmental conditions and is widely used as forage for camels, sheep, and goats. Although it has long been recognized in traditional practices, its phytochemical composition and biological properties have received limited scientific attention. In this study, the phytochemical profile of *S. plumosa* was investigated together with its antioxidant, antimicrobial, and cytotoxic activities. Different solvent extracts were evaluated against pathogenic bacteria, fungi, and clinical veterinary isolates, while their antioxidant capacity and cytotoxic effects on human cancer cell lines were also assessed. Among the tested extracts, the ethyl acetate fraction exhibited the strongest antioxidant and antimicrobial activities, whereas the chloroform fraction showed the highest cytotoxic activity. These findings suggest that *S. plumosa* is a valuable source of naturally occurring bioactive compounds with potential applications in pharmaceutical, veterinary, and food-related fields. The study also highlights the importance of exploring underutilized desert plants as reservoirs of novel biologically active metabolites.

## 1. Introduction

**Figure 1:**
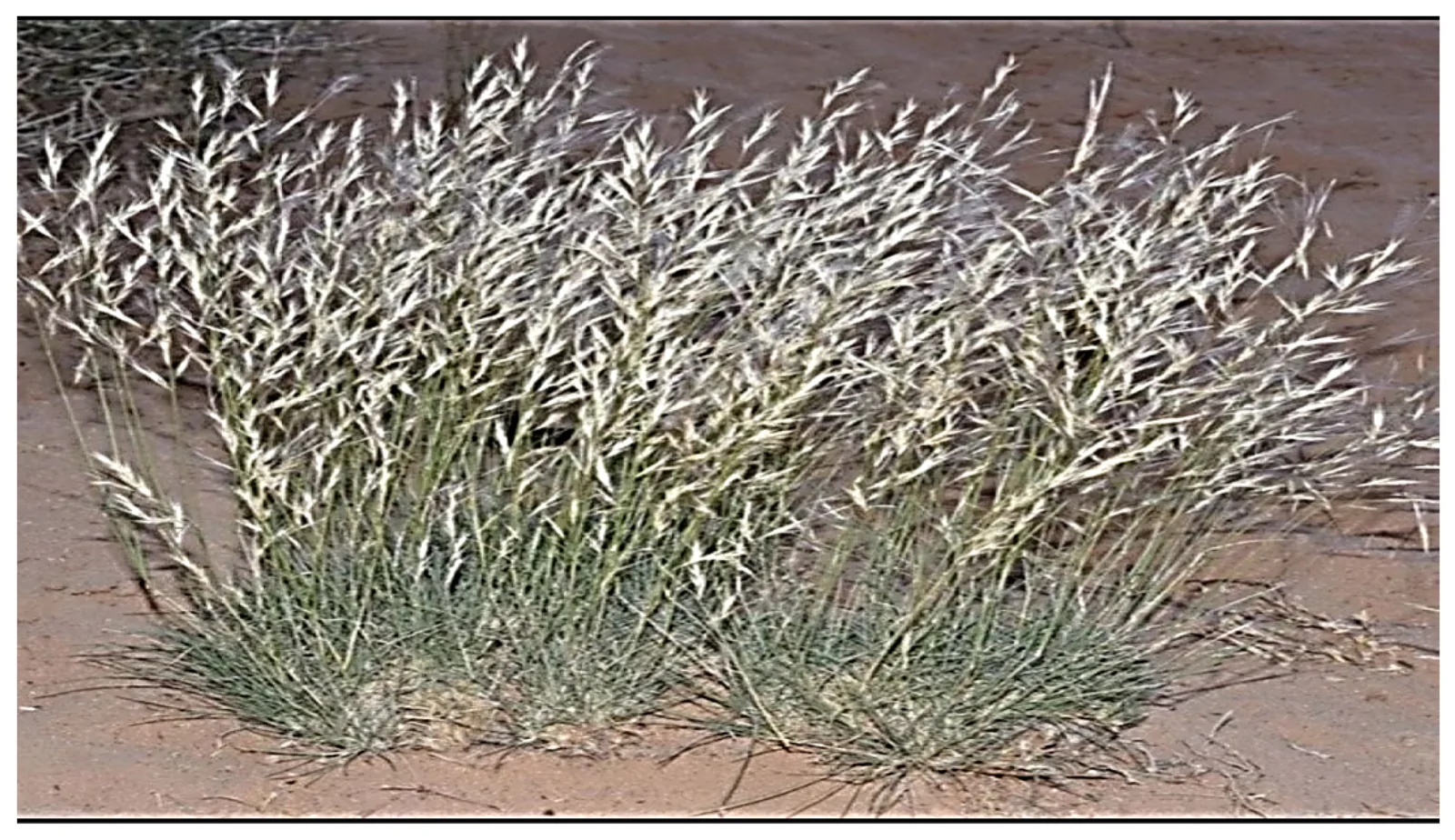
*Stipagrostis plumosa* (Nissi).

*Stipagrostis plumosa* (L.) Munro ex T. Anderson (*Poaceae*) is a perennial desert grass native to arid regions extending from the Sahara across the Arabian Peninsula to parts of Central and South Asia [1] (Figure 1). The species is well adapted to desert and dry shrubland environments and occurs naturally in Saudi Arabia and other countries of the Arabian Peninsula [1]. In Saudi Arabian rangelands, *S. plumosa* contributes to the vegetation of arid ecosystems and is recognized as a palatable forage species for grazing livestock [2,3]. Its pronounced adaptation to drought and other harsh environmental conditions, together with its ecological and forage value, has contributed to scientific interest in this relatively underexplored species [2,3]. Previous phytochemical investigations of the aerial parts of *S. plumosa* have reported phenolic compounds, flavonoids, fatty acids, phytosterols, and other secondary metabolites, as well as antioxidant and cytotoxic activities [4,5]. These findings suggest that *S. plumosa* may represent a promising source of naturally occurring bioactive constituents, although its phytochemical composition and biological activities remain insufficiently characterized compared with those of more extensively studied plant species.

Natural products derived from plants continue to represent an important source of chemically diverse compounds for pharmaceutical and biomedical research. In particular, phenolic acids, flavonoids, and other secondary metabolites have attracted considerable attention because of their diverse biological activities, including antioxidant, antimicrobial, and cytotoxic effects [6,7]. For relatively underexplored plant species, characterization of their phytochemical composition combined with biological evaluation can provide valuable evidence for identifying naturally occurring compounds with potential biomedical applications. Previous investigations of *S. plumosa* have reported phenolic acids, flavonoids, flavolignans, fatty acids, and phytosterols in its aerial parts, together with antioxidant and cytotoxic activities [4,5]. These findings provide a scientific basis for further investigation of *S. plumosa*, particularly to clarify its phytochemical profile and to evaluate its antioxidant, antimicrobial, and cytotoxic properties. Accordingly, the present study was designed to investigate the phytochemical composition and selected biological activities of *S. plumosa* collected from Saudi Arabia, with particular emphasis on its potential as a source of naturally occurring bioactive constituents of relevance to pharmaceutical and veterinary research.

The phytochemical composition and biological properties of *S. plumosa* remain relatively underexplored compared with those of several other members of the *Poaceae* family. For example, *Cymbopogon citratus* has been extensively investigated for its phytochemical constituents, particularly phenolic compounds, flavonoids, and essential-oil components, as well as for a range of biological activities [8]. Similarly, phytochemical and antioxidant investigations of *Cynodon dactylon* have demonstrated the presence of diverse chemical constituents that can vary according to plant part and extraction conditions [9]. *Chrysopogon zizanioides* has also been chemically characterized using chromatographic techniques, including GC–MS, with several constituents reported in association with biological activities [10]. In contrast, information specifically addressing the phytochemical composition and biological activities of *S. plumosa* remains limited, highlighting the need for further systematic investigation of this species.

Recent advances in analytical techniques have facilitated the characterization of plant-derived metabolites and the comparison of chemical profiles among extracts obtained with solvents of different polarities. In particular, GC–MS is widely used for the qualitative characterization of volatile and thermally amenable semi-volatile constituents and can provide valuable information on the chemical diversity of plant extracts [10,11]. Such chemical profiling can support the interpretation of biological activity by identifying candidate constituents for further investigation, although tentative GC–MS library matches should not be considered definitive structural identification without confirmation using authentic standards or complementary analytical techniques. Therefore, characterization of the phytochemical profile of *S. plumosa* may provide a useful basis for investigating its biological properties and identifying candidate compounds of potential pharmaceutical and veterinary interest.

The increasing prevalence of antimicrobial resistance has intensified the search for alternative antimicrobial agents from natural sources. Plant-derived compounds, particularly phenolic acids, flavonoids, terpenoids, and constituents of essential oils, have attracted considerable attention because of their reported ability to inhibit a broad range of pathogenic microorganisms through mechanisms that may include disruption of microbial membranes, interference with essential cellular processes, and modulation of oxidative balance. Several well-studied plant species, including *Thymus vulgaris*, *Origanum vulgare*, and *Rosmarinus officinalis*, have demonstrated antimicrobial activity against diverse bacterial and fungal pathogens, supporting continued investigation of plant-derived metabolites as potential sources of antimicrobial compounds. Although members of the *Poaceae* family have also been investigated for their antimicrobial properties, information concerning the antimicrobial activity of *S. plumosa* remains limited, particularly against microorganisms of veterinary importance. This knowledge gap provides a rationale for evaluating the antimicrobial activity of *S. plumosa* extracts against both reference strains and animal-derived pathogenic isolates [12,13].

In parallel, the antioxidant properties of plant-derived metabolites have received substantial scientific attention because oxidative imbalance can contribute to cellular injury and is implicated in the progression of several pathological conditions. Phenolic compounds and flavonoids can exert antioxidant effects through mechanisms including free-radical scavenging, hydrogen or electron donation, and metal-ion chelation, thereby contributing to protection against oxidative damage [7,14]. Accordingly, evaluation of the antioxidant potential of *S. plumosa*, together with characterization of its major phytochemical constituents, may provide complementary evidence for assessing the biological relevance of this underexplored species.

Despite the growing interest in desert medicinal plants, comprehensive studies integrating phytochemical characterization with biological evaluation of *S. plumosa* remain limited. In particular, little information is available regarding its activity against animal-derived clinical pathogens together with its antioxidant and cytotoxic properties. Therefore, the present study aimed to characterize the phytochemical composition of *S. plumosa* extracts using GC–MS and to evaluate their antimicrobial activity against both reference microorganisms and animal-derived clinical isolates, in addition to assessing their antioxidant and cytotoxic activities. The findings of this study are expected to provide scientific evidence supporting the pharmaceutical and veterinary potential of this underexplored desert grass.

## 2. Materials and Methods

### 2.1. Materials and Reagents

All solvents and analytical-grade reagents required for the experiments were purchased from Sigma-Aldrich (St. Louis, MO, USA). Following extraction, the individual solvent fractions were concentrated separately by rotary evaporation under controlled conditions (40 °C, 200 mbar, and 45 rpm). The resulting dried fractions were placed in sterile amber glass vials and stored at −20 °C until further phytochemical and biological analyses.

Phytochemical profiling was conducted using an Agilent 7890B gas chromatography system coupled to a mass spectrometer (Agilent Technologies, Santa Clara, CA, USA). Compound identification was based on comparison of the obtained mass spectra with entries in the NIST Mass Spectral Library. The A549 human lung adenocarcinoma and MCF-7 human breast adenocarcinoma cell lines were obtained from the DSMZ–Leibniz Institute (German Collection of Microorganisms and Cell Cultures, Braunschweig, Germany).

### 2.2. Bacterial and Fungal Strains

The antimicrobial properties of *S. plumosa* extracts were investigated using both established reference strains and clinical isolates obtained from animals. The animal-derived isolates comprised *Klebsiella pneumoniae*, *Enterococcus faecium*, *Pseudomonas aeruginosa*, *Staphylococcus aureus*, *Escherichia coli*, *Salmonella Typhimurium*, *Bacillus cereus*, *Streptococcus pyogenes*, and *Candida albicans*. These isolates were collected from clinically healthy and diseased animals and poultry maintained on livestock and poultry farms in Giza, Egypt.

For comparison, the reference microorganisms were procured from Sigma-Aldrich (St. Louis, MO, USA) and consisted of *Enterococcus faecalis* ATCC 29212, *Salmonella Typhimurium* ATCC 14028, *Escherichia coli* ATCC 25922, *Staphylococcus aureus* ATCC 29213, *Candida tropicalis* ATCC 66029, and *Candida albicans* ATCC 60193.

### 2.3. Plant Material Collection and Preparation

#### 2.3.1. Collection of Plant Material

The aerial portions of *S. plumosa* were collected at the flowering stage in April 2022 from Wadi Al-Quwai’iyah, Al Quwai’iyah Governorate, Riyadh Region, Saudi Arabia (latitude: 24.0670° N, longitude: 45.2806° E). The sampling site represents an arid desert environment characterized by natural vegetation adapted to water-limited conditions, including drought-tolerant grasses and shrubs that contribute to the available grazing resources. *S. plumosa* occurs as a perennial grass within this vegetation and has been traditionally used as livestock forage and in local folk medicine. After collection, the plant material was transferred to the laboratory for botanical authentication and subsequent phytochemical and biological analyses.

#### 2.3.2. Plant Identification and Authentication

Botanical identification of the collected specimens was carried out by Dr. Mashail Nasser Al-Zain, Assistant Professor of Plant Ecology, Department of Biology, Princess Nourah bint Abdulrahman University, Riyadh, Saudi Arabia. The taxonomic identity was further authenticated by comparison with a verified herbarium specimen deposited at the Herbarium of the College of Pharmacy, King Saud University, Riyadh, Saudi Arabia (Voucher No. 15889).

### 2.4. Extraction Processes

#### 2.4.1. Aqueous Extraction

Aqueous extracts of the powdered aerial material of *S. plumosa* were prepared using cold- and hot-water extraction procedures. For each extraction, 200 g of the powdered plant material was mixed with distilled water at a 1:10 (*w*/*v*) ratio. For the cold-water extraction, the mixture was maintained at 25 ± 2 °C for 24 h with intermittent shaking. For the hot-water extraction, distilled water preheated to 80–90 °C was added to the plant material, and extraction was continued for 30 min. The extracts were subsequently filtered through Whatman No. 1 filter paper, concentrated under reduced pressure, freeze-dried when required, and stored at 4 °C in amber bottles until further analysis [15].

#### 2.4.2. Organic Solvent Extraction

Organic extraction was carried out by conventional maceration using 80% methanol, following the procedure reported for the recovery of phytochemicals from medicinal plants [15]. Briefly, the air-dried aerial parts of *S. plumosa* were milled to obtain a coarse powder. A 200 g portion of the powdered material was immersed in 500 mL of 80% (*v*/*v*) methanol and macerated at room temperature (25 ± 2 °C) for 48–72 h with intermittent shaking. The extraction was repeated several times until exhaustive extraction was achieved. The resulting extracts were pooled and filtered through Whatman No. 1 filter paper (approximately 11 µm pore size). The filtrate was concentrated under reduced pressure using a rotary evaporator (BÜCHI R-205, Labortechnik AG, Flawil, Switzerland) at 40 °C, 200 mbar, and 45 rpm until the solvent was completely removed. The resulting crude methanolic extract was resuspended in distilled water and subjected to sequential liquid–liquid partitioning with n-hexane, chloroform, ethyl acetate, diethyl ether, acetone, and n-butanol. Each solvent partition was performed in three successive extraction steps using 200 mL of solvent per step. The resulting fractions were individually concentrated under the same rotary evaporation conditions, transferred to amber glass vials, and stored at −20 °C in the absence of light until phytochemical and biological analyses.

### 2.5. Phytochemical Analysis

Phytochemical quantification was performed on the crude methanolic extract to determine its total phenolic and flavonoid contents. The crude methanolic extract was selected for this analysis because it was obtained before solvent partitioning and was therefore considered representative of the overall pool of extractable polar phytochemicals. The subsequent solvent fractions were primarily investigated to assess differences in biological activity associated with solvent polarity rather than to quantify their individual phenolic and flavonoid contents.

#### 2.5.1. Determination of Total Phenolic Content

The total phenolic content (TPC) of the crude methanolic extract of *S. plumosa* was assessed using the Folin–Ciocalteu colorimetric method following the procedure of Do et al. [16]. Briefly, a measured aliquot of the extract was reacted with Folin–Ciocalteu reagent, followed by the addition of sodium carbonate solution. The reaction mixture was incubated at room temperature, and its absorbance was subsequently determined spectrophotometrically at the specified wavelength. A calibration curve was prepared using gallic acid as the reference standard. TPC was expressed as milligrams of gallic acid equivalents per gram of dry extract (mg GAE/g extract).

#### 2.5.2. Determination of Total Flavonoid Content

The total flavonoid content (TFC) of the crude methanolic extract was quantified using an aluminum chloride-based colorimetric assay following the method reported by Chang et al. [17], with minor modifications. An aliquot of the extract was combined with the aluminum chloride reagent and maintained at 25 ± 2 °C for 30 min to facilitate development of the flavonoid–aluminum complex. The absorbance of the resulting reaction mixture was subsequently recorded at the appropriate wavelength using a UV–visible spectrophotometer. Quercetin was used as the reference standard for calibration, and the TFC was expressed as milligrams of quercetin equivalents per gram of dry extract (mg QE/g extract).

#### 2.5.3. Gas Chromatography–Mass Spectrometry (GC–MS) Analysis

GC–MS was employed to characterize the chemical constituents present in the ethyl acetate and chloroform extracts of *S. plumosa*, according to the procedure reported by Porras et al. [18]. The analyses were conducted using an Agilent Technologies GC–MS system fitted with a DB-5MS capillary column (30 m × 0.25 mm i.d.; 0.25 μm film thickness). Helium served as the carrier gas and was maintained at a constant flow rate of 1.0 mL/min. The injector was operated at 250 °C with a split ratio of 50:1, while the oven was programmed from 50 to 250 °C over a total run time of 50 min.

Mass spectra were recorded within an *m*/*z* range of 40–500, using a solvent delay of 9 min and an ion source temperature of 230 °C. Putative compound assignments were made by matching the acquired mass spectra against entries in the NIST Mass Spectral Library. Compounds were retained in the final dataset only when their spectral matching score was ≥80% and their identities were considered compatible with the expected phytochemical composition of the plant. Signals attributed to common analytical contaminants, such as siloxanes and phthalates, as well as other laboratory-related artifacts, were excluded from subsequent biological interpretation.

### 2.6. Biological Assays

#### 2.6.1. Antioxidant Activity

The antioxidant capacity of the *S. plumosa* extracts and their respective fractions was assessed by the ABTS radical cation scavenging assay following the procedure reported by AlZain et al. [19]. The ABTS radical solution was generated by allowing ABTS to react with the designated oxidizing agent. The prepared radical solution was subsequently combined with aliquots of the test extracts, and the resulting change in absorbance was determined spectrophotometrically at the appropriate wavelength. Antioxidant activity was quantified in terms of the IC_50_ value, defined as the extract concentration required to achieve 50% scavenging of the ABTS radical cations.

#### 2.6.2. Cell Viability Assay

The effects of *S. plumosa* extracts on the viability of A549 and MCF-7 cells were examined using the MTT colorimetric assay, following the procedure reported by AlZain et al. [19]. Briefly, the cells were dispensed into 96-well plates and treated with the tested extracts at different concentrations. After the specified incubation period, MTT reagent was added to the wells and the plates were further incubated to permit the formation of formazan crystals by viable metabolically active cells. The resulting crystals were subsequently solubilized using the appropriate solution, and absorbance was recorded at 570 nm with a microplate reader. Cell viability was determined in comparison with untreated control cells and expressed as a percentage.

#### 2.6.3. Antimicrobial Activity Screening (Agar Well-Diffusion Assay)

A two-step approach was adopted to characterize the antimicrobial activity of *S. plumosa* extracts. Initially, the agar well-diffusion assay was applied to screen the different solvent extracts and compare their inhibitory effects based on the diameter of the resulting inhibition zones. Extracts showing the strongest activity in this preliminary screening were subsequently selected for quantitative assessment by broth microdilution to establish their MIC and MBC/MFC values.

The agar well-diffusion assay was conducted following the procedure reported by AlZain et al. [19]. Bacterial and fungal inocula were standardized to a turbidity equivalent to 0.5 McFarland before being spread onto Mueller–Hinton agar and Sabouraud dextrose agar, respectively. Wells measuring 6 mm in diameter were aseptically prepared using a cork borer, and 100 µL of each extract was added to the designated wells. Sterile distilled water was included as the negative control, while ciprofloxacin and nystatin served as positive controls for bacterial and fungal assays, respectively. The inoculated plates were incubated under the appropriate conditions for the microorganisms tested, after which the inhibition zones were measured in millimeters. Each assay was conducted in triplicate, and the results were expressed as mean ± SD.

#### 2.6.4. Determination of Minimum Inhibitory Concentration (MIC)

Following the preliminary screening by agar well diffusion, the ethyl acetate, crude methanolic, and chloroform extracts, which exhibited the greatest inhibitory effects, were subjected to quantitative antimicrobial assessment using the broth microdilution technique described by AlZain et al. [19]. Serial two-fold dilutions of each selected extract were prepared at concentrations ranging from 5 to 150 mg/mL in sterile 96-well microplates containing Mueller–Hinton broth for bacterial testing or Sabouraud dextrose broth for fungal testing. Each dilution was inoculated with a standardized microbial suspension, while appropriate growth and sterility controls were included in each assay. After incubation under the conditions appropriate for the respective microorganisms, the wells were examined for visible microbial growth. The MIC was recorded as the lowest extract concentration at which no visible growth was observed.

#### 2.6.5. Determination of Minimum Bactericidal/Fungicidal Concentration (MBC/MFC)

The bactericidal and fungicidal endpoints were determined by further processing the wells identified as negative for visible growth in the MIC assay. A 10 µL aliquot from each well showing no visible microbial growth was subcultured onto Mueller–Hinton agar for bacterial isolates and Sabouraud dextrose agar for fungal isolates, followed by incubation under the appropriate conditions. The MBC or MFC was defined as the lowest extract concentration at which no visible microbial colonies developed following subculture, indicating the absence of recoverable viable microorganisms. The procedure was performed according to AlZain et al. [19].

### 2.7. Statistical Analysis

Experimental data obtained from the assays performed in triplicate were expressed as mean ± standard deviation (SD). Statistical comparisons were conducted using one-way analysis of variance (ANOVA) in OriginPro 8.5. Statistical significance was set at *p* ≤ 0.05. Correlation analysis was performed using SAS software (v9.4, SAS Institute Inc., Cary, NC, USA) according to the procedure described by AlZain et al. [19].

## 3. Results

The phytochemical composition and biological activities of *S. plumosa* extracts were investigated through quantitative phytochemical analysis, GC–MS profiling, and in vitro antioxidant, antimicrobial, and cytotoxic assays. The obtained findings are presented in the following sections.

### 3.1. Total Phenolic and Flavonoid Contents

The total phenolic and flavonoid contents of the crude methanol extract of *S. plumosa* are presented in Table 1. The extract contained 72.1 mg GAE/g dry extract of total phenolics and 13.6 mg QE/g dry extract of total flavonoids.

### 3.2. GC-MS Profile of Stipagrostis plumosa

The phytochemical profiles of the ethyl acetate and chloroform extracts of *S. plumosa* were investigated using GC–MS. The chromatographic analyses revealed complex mixtures containing compounds belonging to different chemical classes, including phenolic compounds, fatty acids and their esters, phytosterol-related constituents, and oxygenated heterocyclic compounds. Differences in the composition and relative abundance of the detected constituents were observed between the two extracts, reflecting the selectivity of the extraction solvents.

The GC–MS library-search output contained 52 peak entries for the ethyl acetate extract, of which 50 yielded tentative library assignments, corresponding to 96.15% of the reported peak entries. For the chloroform extract, 95 peak entries were reported, of which 94 yielded tentative library assignments, corresponding to 98.95% of the reported peak entries. The assignments were based on comparison of the obtained mass spectra with reference spectra available in the NIST mass spectral library and should therefore be considered tentative rather than confirmed compound identifications. Peak numbering follows the original GC–MS analytical output; therefore, occasional gaps in the numbering are retained. The complete GC–MS library-search outputs, including retention time, peak area, tentative library hit, library-match quality, molecular weight, and CAS number where available, are provided in Supplementary Tables S1 and S2. The corresponding complete chromatographic profiles are presented in Supplementary Figures S1 and S2.

In the ethyl acetate extract, the major tentatively assigned constituents selected for presentation in the main text included stigmasta-3,5-dien-7-one (14.70%), vanillic acid (7.18%), 2-pentadecanone, 6,10,14-trimethyl- (5.83%), 2-methoxy-4-vinylphenol (4.77%), dodecanoic acid (4.77%), and tetradecanoic acid (4.39%). Other constituents selected for presentation included 4,8,12,16-tetramethylheptadecan-4-olide (2.88%) and vanillin (2.51%). These compounds showed relatively high library-match quality scores, ranging from 90 to 99, in the analytical output.

The chloroform extract showed a different distribution of major tentatively assigned constituents. The most abundant compounds selected for presentation included hexadecanoic acid, methyl ester (5.28%), ethyl 4-hydroxy-3-methoxybenzoate [Benzoic acid, 4-hydroxy-3-methoxy-, ethyl ester] (5.22%), 6-hydroxy-4,4,7a-trimethyl-5,6,7,7a-tetrahydrobenzofuran-2(4H)-one (4.67%), tetradecanoic acid (4.30%), octadecanoic acid (3.71%), and dodecanoic acid (2.77%). Other relatively abundant constituents included 4,8,12,16-tetramethylheptadecan-4-olide (3.17%) and α-tocospiro A (1.31%). The corresponding library-match quality scores for these selected compounds ranged from 83 to 99.

For interpretation of the biological relevance of the GC–MS results, emphasis was placed on compounds with relatively high relative peak abundance, acceptable library-match quality, and plausible occurrence in a plant-derived extract. Compounds giving low-quality or ambiguous library matches, as well as putative contaminants, derivatization-related products, and compounds with questionable phytochemical plausibility, were not considered in the biological interpretation. This precaution was particularly important because the complete analytical output contained several library hits suggestive of phthalates, siloxane-related compounds, and other non-plant or analytically derived substances. These entries were retained in the Supplementary Materials to ensure transparency of the analytical dataset but were excluded from the biological interpretation.

Accordingly, Table 2 presents only the major tentatively assigned compounds selected for biological discussion, rather than the complete GC–MS profile. The cumulative peak-area values shown in Table 2 represent only the sum of the relative peak areas of the selected major compounds and therefore should not be interpreted as the percentage of all compounds identified in either extract. The complete analytical datasets are provided in Supplementary Tables S1 and S2, while the complete chromatograms are provided in Supplementary Figures S1 and S2.

Only major compounds with relatively high abundance, acceptable spectral matching quality (≥80%), and plausible phytochemical origin are presented in Table 2. The cumulative peak area values therefore represent only the compounds selected for presentation and do not represent the total chromatographic area of each extract. A dash (−) indicates that the compound was not detected in the corresponding extract. The complete GC–MS analytical datasets are provided in Supplementary Tables S1 and S2.

### 3.3. Antioxidant Scavenging Activity

**Figure 2:**
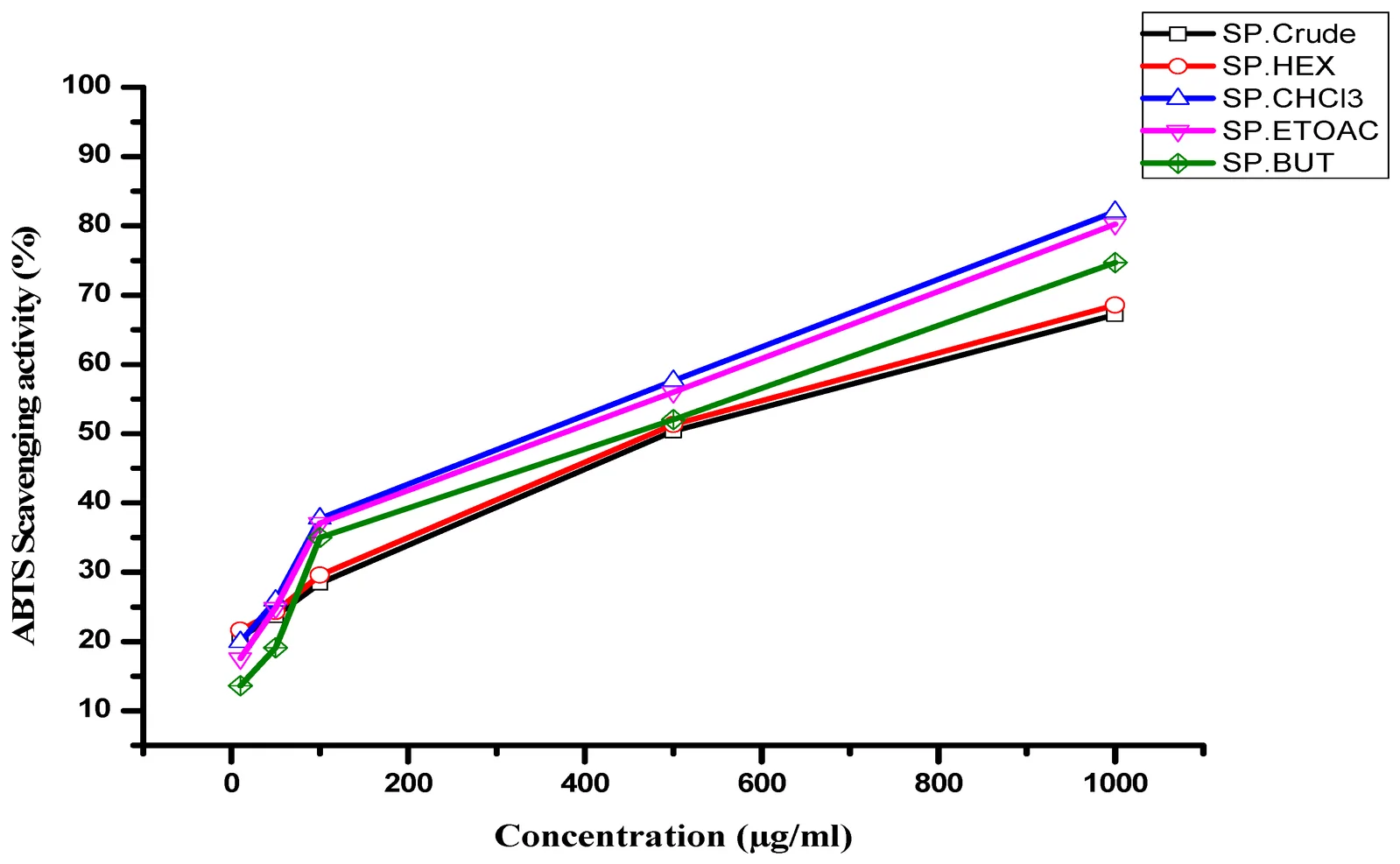
Antioxidant activity of the crude methanol extract and solvent fractions of *Stipagrostis plumosa* determined using the ABTS radical scavenging assay. Abbreviations: Crude, crude methanol extract; Hex, *n*-hexane fraction; CHCl_3_, chloroform fraction; EtOAc, ethyl acetate fraction; BUT, *n*-butanol fraction.

The antioxidant activity of *S. plumosa* extracts and solvent fractions was evaluated using the ABTS radical scavenging assay, and the results are shown in Figure 2. Antioxidant activity increased in a concentration-dependent manner for all tested extracts. Based on the IC_50_ values, the ethyl acetate fraction exhibited the highest antioxidant activity (IC_50_ = 72.48 µg/mL), followed by the chloroform and *n*-butanol fractions. The crude methanol extract showed lower antioxidant activity (IC_50_ = 369.80 µg/mL), whereas quercetin, used as the positive control, exhibited the strongest radical scavenging activity (IC_50_ = 1.70 µg/mL).

### 3.4. Cytotoxic Activity

**Figure 3:**
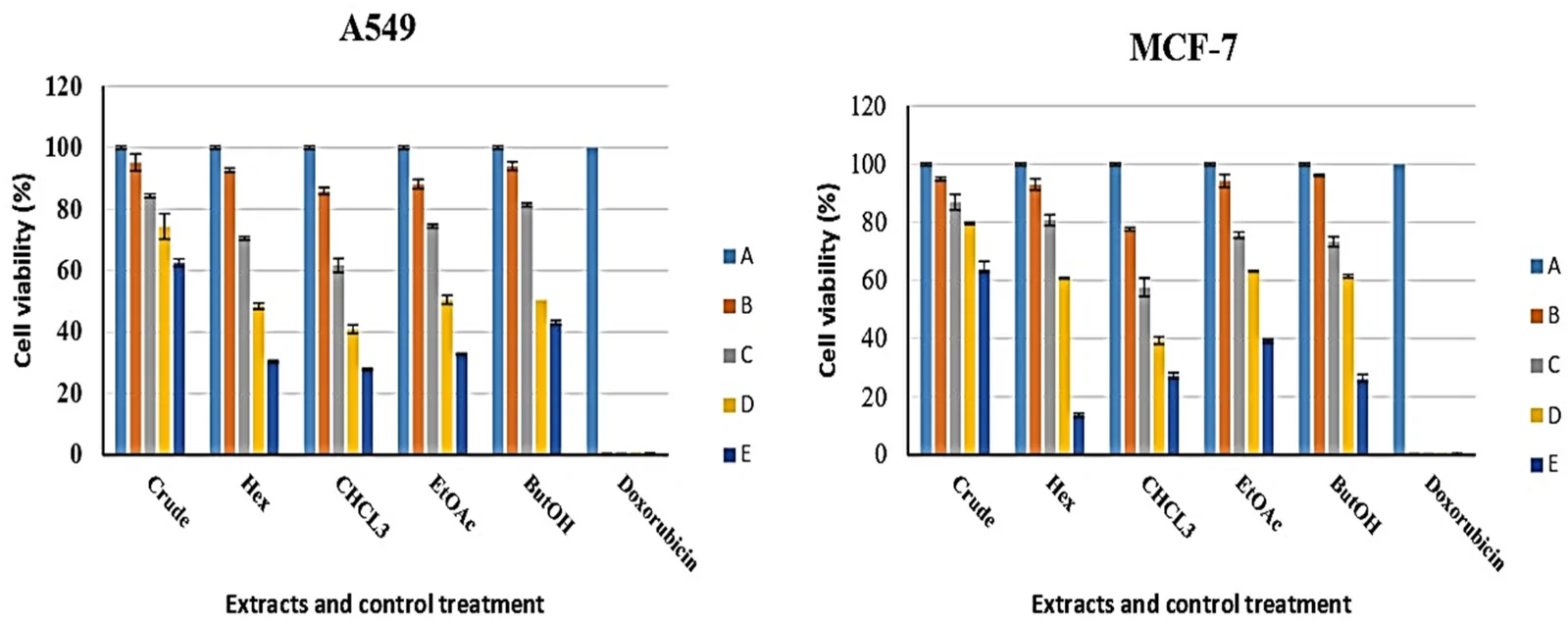
Cytotoxic activity of the crude methanol extract and solvent fractions of *Stipagrostis plumosa* against MCF-7 and A549 cell lines, as determined by the MTT assay. Cells were exposed to different concentrations (A = 0 µg/mL, B = 62.5 µg/mL, C = 125 µg/mL, D = 250 µg/mL, and E = 500 µg/mL), and cell viability was expressed as a percentage relative to the untreated control. Doxorubicin was used as the positive control. Data are presented as mean ± SD from three independent experiments (*n* = 3).

The cytotoxic activity of the crude methanol extract and solvent fractions of *S. plumosa* was evaluated against A549 and MCF-7 cancer cell lines using the MTT assay. The IC_50_ values are summarized in Table 3, while the dose-dependent responses are illustrated in Figure 3. Among the tested fractions, the chloroform fraction exhibited the strongest cytotoxic activity against both cell lines, with IC_50_ values of 194.17 ± 2.5 µg/mL for A549 cells and 173.52 ± 2.7 µg/mL for MCF-7 cells. The hexane fraction showed moderate cytotoxic activity, followed by the ethyl acetate and *n*-butanol fractions. No detectable cytotoxic activity was observed for the crude methanol extract under the tested conditions. As expected, doxorubicin, used as the positive control, exhibited the highest cytotoxic activity, with IC_50_ values of 3.52 ± 0.05 µg/mL and 2.50 ± 0.03 µg/mL against A549 and MCF-7 cells, respectively.

### 3.5. Antimicrobial Activity

The antimicrobial activities of the crude methanol extract and solvent fractions of *S. plumosa* against the tested bacterial and fungal strains are presented in Table 4, Table 5 and Table 6. The antimicrobial responses varied according to the extraction solvent. Among the tested extracts, the ethyl acetate fraction exhibited the highest antimicrobial activity against both Gram-positive and Gram-negative bacteria, as well as the tested fungal strains. The crude methanol and chloroform extracts also demonstrated marked antimicrobial activity, whereas the *n*-hexane, *n*-butanol, and ether extracts showed moderate inhibitory effects. In contrast, the acetone and cold aqueous extracts exhibited relatively weak antimicrobial activity, while the hot aqueous extract showed the lowest activity, with little or no antifungal effect. Statistical analysis revealed significant differences among the tested extracts (*p* < 0.05).

### 3.6. Antimicrobial Activity: MIC, MBC, and MFC

Following preliminary screening by the agar well-diffusion assay, the ethyl acetate, crude methanolic, and chloroform extracts were selected for quantitative evaluation using the broth microdilution method. The MIC and MBC/MFC values obtained against the tested reference strains and animal-derived isolates are summarized in Table 6. The ethyl acetate extract generally showed the highest antimicrobial potency, as indicated by the lowest MIC and MBC/MFC values against most of the tested microorganisms, followed by the crude methanolic and chloroform extracts. The MIC and MBC/MFC endpoints were determined in three independent experiments, with identical concentrations obtained across the replicates (SD = 0.00).

## 4. Discussion

The present study provides an integrated evaluation of the phytochemical composition and biological properties of *S. plumosa* collected from Saudi Arabia. The study combined quantitative determination of total phenolic and flavonoid contents, antioxidant assessment, antimicrobial evaluation against standard strains and animal-derived pathogenic isolates, cytotoxicity testing against MCF-7 and A549 cancer cell lines, and GC–MS profiling of the ethyl acetate and chloroform fractions. Taken together, the results demonstrate that *S. plumosa* contains chemically diverse constituents associated with measurable antioxidant, antimicrobial, and cytotoxic activities. However, the biological effects observed in the present study should be interpreted as activities of complex extracts rather than as evidence for the therapeutic efficacy of the plant or any individual compound.

The crude methanolic extract contained 72.1 mg GAE/g extract of total phenolic compounds and 13.6 mg QE/g extract of total flavonoids. These values indicate a measurable contribution of phenolic constituents to the chemical composition of the crude extract. Phenolic compounds can contribute to antioxidant activity through hydrogen- or electron-donation mechanisms, radical stabilization, and interactions with redox-active metal ions. However, the relationship between total phenolic content and antioxidant activity is not necessarily linear because the Folin–Ciocalteu assay measures the overall reducing capacity of an extract rather than the concentration of individual phenolic molecules [20,21].

The TPC obtained in the present study can be placed within the range previously reported for *S. plumosa*, although the values are not directly comparable because of differences in extraction procedures, plant material, geographical origin, and expression of results. Abdelkader et al. reported TPC values ranging from 10.44 to 42.33 mg GAE/g for different fractions of *S. plumosa*, with the ethyl acetate fraction showing the highest value (42.33 ± 0.57 mg GAE/g) [4]. The higher value obtained in the present crude methanolic extract (72.1 mg GAE/g) may therefore reflect differences in extraction efficiency and sample characteristics rather than an intrinsic difference in phenolic richness between populations. In another study of the closely related species *Stipagrostis ciliata*, TPC values ranged from 34.45 ± 0.68 to 263.16 ± 5.26 mg GAE/g depending on solvent, with methanol producing the highest value, while the ethyl acetate fraction contained 70.93 ± 1.41 mg GAE/g [22]. These findings reinforce the importance of solvent polarity and extraction strategy when comparing phenolic contents among *Stipagrostis* species.

The TFC value obtained in the present study (13.6 mg QE/g extract) also falls within the range reported for related *Stipagrostis* material. Iram et al. reported flavonoid contents of approximately 29.5 and 29.2 mg QE/g for *S. plumosa* extracts obtained using different extraction conditions, whereas *S. ciliata* exhibited TFC values ranging from 7.56 ± 0.03 to 107.21 ± 0.53 mg QE/g depending on the solvent [22,23]. Such differences emphasize that TPC and TFC values should not be considered intrinsic fixed characteristics of a species. Instead, they are strongly affected by solvent polarity, extraction procedure, plant part, geographical origin, environmental conditions, and the analytical assay employed.

An important finding of the present study was that the ethyl acetate fraction exhibited the strongest antioxidant activity, with an IC_50_ of 72.48 µg/mL, despite the fact that TPC and TFC were quantified only in the crude methanolic extract. This result does not necessarily indicate a contradiction between the phenolic-content measurements and antioxidant activity. During liquid–liquid fractionation, compounds with different polarities are redistributed among the fractions, and moderately polar constituents may become enriched in ethyl acetate. Previous investigations have similarly demonstrated that ethyl acetate fractions can concentrate antioxidant constituents and may display stronger radical-scavenging activity than the corresponding crude extracts [16,24]. In *S. plumosa*, Abdelkader et al. likewise reported that the ethyl acetate fraction exhibited the strongest DPPH-scavenging activity among the tested fractions [4]. Therefore, the strong antioxidant activity of the ethyl acetate fraction in the present study may reflect selective enrichment of antioxidant-active constituents during fractionation. Nevertheless, because TPC and TFC were not determined separately for all fractions in the present work, a direct statistical relationship between phenolic/flavonoid content and antioxidant activity cannot be established. This distinction is important when interpreting the antioxidant results.

The GC–MS profiles further demonstrated marked differences in the chemical composition of the ethyl acetate and chloroform fractions. The ethyl acetate fraction was particularly characterized by Stigmasta-3,5-dien-7-one (14.70%), vanillic acid (7.18%), 2-pentadecanone, 6,10,14-trimethyl- (5.83%), 2-methoxy-4-vinylphenol (4.77%), dodecanoic acid (4.77%), and tetradecanoic acid (4.39%). The chloroform fraction, in contrast, contained relatively high proportions of hexadecanoic acid methyl ester (5.28%), a hydroxy-methoxy benzoate derivative (5.22%), 6-hydroxy-4,4,7a-trimethyl-5,6,7,7a-tetrahydrobenzofuran-2(4H)-one (4.67%), tetradecanoic acid (4.30%), octadecanoic acid (3.71%), and dodecanoic acid (2.77%). The different chemical patterns observed between the two fractions are consistent with solvent-dependent partitioning of plant metabolites.

The detection of phenolic constituents provides a plausible chemical basis for part of the antioxidant activity observed in the present study. Vanillic acid and vanillin were detected in the ethyl acetate fraction, while vanillic acid and related aromatic derivatives were also detected in the chloroform fraction. Vanillin and vanillic acid have been reported to possess antioxidant and antimicrobial properties, although their biological effects depend strongly on concentration and experimental conditions [25]. In particular, vanillic acid has demonstrated radical-scavenging activity and antibacterial effects, while studies of its antibacterial mechanism have implicated changes in bacterial physiological and membrane-related parameters [26]. Similarly, 2-methoxy-4-vinylphenol, which represented 4.77% of the ethyl acetate fraction, has been associated with antioxidant and antimicrobial activities in other plant-derived extracts [27]. These observations support the possibility that phenolic constituents contributed to the antioxidant and antimicrobial effects of the ethyl acetate fraction. However, because the GC–MS assignments are tentative library-based identifications, these compounds should be regarded as putatively identified rather than unequivocally confirmed constituents.

The relatively high abundance of fatty acids and their derivatives in both fractions may also be relevant to the antimicrobial activity. Dodecanoic acid, tetradecanoic acid, and octadecanoic acid were detected in the two fractions, together with several fatty acid esters. Fatty acids have been reported to exert antimicrobial effects through several mechanisms, including interactions with microbial membranes and interference with essential metabolic processes. Unsaturated fatty acids may additionally inhibit bacterial fatty-acid biosynthesis by targeting FabI, an essential enzyme in bacterial lipid metabolism [28]. The presence of these compounds may therefore contribute to the broad antimicrobial activity observed in the present study. Nevertheless, the contribution of individual fatty acids cannot be established from the present GC–MS data alone because the biological assays were performed using complex fractions rather than isolated compounds.

The antimicrobial results showed that the ethyl acetate fraction generally exhibited the strongest activity against the tested microorganisms, as reflected by inhibition zones and the corresponding MIC and MBC/MFC results. Importantly, activity was observed against both Gram-positive and Gram-negative bacteria as well as *Candida* species. The activity against animal-derived pathogenic isolates is particularly relevant because it extends the biological evaluation beyond standard laboratory strains. However, the results should be interpreted as preliminary in vitro evidence of antimicrobial potential rather than as evidence of therapeutic efficacy. The stronger activity of the ethyl acetate fraction may be associated with its enrichment in phenolic constituents and other moderately polar metabolites, while fatty acids and their derivatives may provide additional membrane-active effects. Similar solvent-dependent antimicrobial patterns have been described for plant fractions in which medium-polarity fractions contained higher concentrations of bioactive phenolics and showed enhanced antimicrobial activity [29].

The relationship between antioxidant and antimicrobial activity observed in the present study may reflect partially overlapping chemical determinants. Phenolic compounds can interfere with microbial cell membranes and proteins, whereas their redox properties may contribute to oxidative damage within microbial cells. Vanillin, for example, has been reported to interact with microbial membranes and membrane-associated components, providing a plausible explanation for antimicrobial activity of phenolic-rich extracts [25]. Nevertheless, the strong antimicrobial activity of a plant fraction cannot be attributed exclusively to its antioxidant capacity, because antioxidant and antimicrobial assays measure fundamentally different biological phenomena. The observed activity is more appropriately considered a consequence of the combined effects of several constituents present within each fraction.

The cytotoxicity results showed that the chloroform fraction exhibited the strongest activity against both MCF-7 breast cancer and A549 lung cancer cells, with IC_50_ values of 173.52 and 194.17 µg/mL, respectively. These values indicate moderate in vitro cytotoxic activity and are considerably less potent than the positive anticancer control used in the experiment. The result is nevertheless consistent with previous investigations of *S. plumosa*. Abdelkader et al. reported moderate to low cytotoxic activities for fractions and isolated compounds from the species, with IC_50_ values generally ranging from 120 to 670 µg/mL, while Hussein et al. subsequently demonstrated cytotoxic activity of *S. plumosa* extracts and isolated phenolic constituents against several human cancer cell lines, including A549 and MCF-7 [4,5]. Thus, the present findings provide additional evidence that the species contains constituents capable of affecting cancer-cell viability in vitro.

The relatively stronger cytotoxicity of the chloroform fraction may be associated with its different chemical profile. This fraction contained several relatively abundant fatty acid derivatives, aromatic compounds, and oxygenated metabolites that were not present at comparable abundance in the ethyl acetate fraction. In addition, vanillin and vanillic acid were detected in the chloroform profile. Previous studies have demonstrated that vanillin can inhibit proliferation of MCF-7 and A549 cells and has been associated with reduced CDK6 expression and induction of apoptosis [30]. Vanillic acid has also been investigated for antiproliferative and pro-apoptotic effects through mechanisms involving cell-cycle regulation, apoptosis-related pathways, and signaling pathways such as PI3K/AKT/mTOR and NF-κB [31]. These findings provide plausible biological mechanisms through which aromatic phenolic constituents could contribute to the cytotoxic activity observed in the present chloroform fraction. However, such mechanisms should be regarded as hypotheses generated from the chemical profile and previous literature, rather than mechanisms demonstrated experimentally in the present study.

The present findings also highlight the importance of considering the combined effects of multiple constituents. Plant extracts contain complex mixtures in which phenolic acids, phenolic aldehydes, fatty acids, sterol derivatives, and other metabolites may act independently or interact additively or synergistically. The biological activity of an extract therefore cannot be predicted solely from the abundance of its single major GC–MS peak. This is particularly relevant to the ethyl acetate fraction, in which Stigmasta-3,5-dien-7-one represented 14.70% of the chromatographic area but cannot by itself be considered responsible for the observed antioxidant or antimicrobial activity. Previous phytochemical work on *S. plumosa* has demonstrated the occurrence of several phenolic compounds, including tricin derivatives and phenolic acids, and has shown antioxidant and cytotoxic activities associated with extracts and isolated compounds [5]. The present GC–MS results therefore complement rather than replace previous phytochemical investigations by demonstrating a distinct set of volatile/semi-volatile and derivatizable constituents in the tested fractions.

An additional point that should be considered is the difference between the present GC–MS profile and previous phytochemical investigations of *S. plumosa*. Hussein et al. reported ten isolated phenolic compounds, including tricin derivatives, luteolin glycosides, apigenin derivatives, and phenolic acids, in addition to 29 non-polar compounds identified by GC–MS [5]. In contrast, the present study identified a broader set of tentative GC–MS hits across the ethyl acetate and chloroform fractions. Such differences are expected because the extraction solvents, analytical platforms, plant material, geographical origin, and chromatographic conditions differed between studies. The present results should therefore be interpreted as a solvent-specific chemical profile rather than a complete representation of the metabolome of *S. plumosa*.

The antioxidant, antimicrobial, and cytotoxic results collectively suggest that solvent fractionation substantially influenced the biological properties of the extracts. The ethyl acetate fraction showed the strongest antioxidant and antimicrobial performance, whereas the chloroform fraction displayed the greatest cytotoxic activity against MCF-7 and A549 cells. This divergence is scientifically informative because it indicates that the metabolites responsible for different biological effects may not be distributed uniformly among the fractions. Such solvent-dependent bioactivity provides a useful basis for future bioassay-guided fractionation aimed at identifying the individual constituents responsible for each activity.

The present study has several limitations that should be considered when interpreting its biological significance. First, TPC and TFC were determined only for the crude methanolic extract; therefore, direct correlations between phenolic/flavonoid content and the biological activities of individual fractions cannot be established. Second, the GC–MS identifications are tentative and based primarily on spectral-library matching; structural confirmation using authentic standards and complementary spectroscopic techniques would be required to establish the identity of individual constituents conclusively. Third, the antioxidant, antimicrobial, and cytotoxic assays were conducted in vitro, and the results do not establish pharmacological efficacy, selectivity, bioavailability, or safety in vivo. Finally, the biological activity of a complex fraction cannot be attributed to individual compounds solely on the basis of their relative GC–MS abundance. These limitations do not diminish the value of the findings but define the appropriate level of interpretation.

Overall, the present study expands the limited experimental literature on *S. plumosa* by demonstrating that Saudi Arabian material contains chemically diverse metabolites and exhibits measurable antioxidant, antimicrobial, and cytotoxic activities. The stronger antioxidant and antimicrobial activity of the ethyl acetate fraction and the comparatively greater cytotoxic activity of the chloroform fraction demonstrate that biological activity is strongly dependent on solvent fractionation and chemical composition. The findings support further investigation of *S. plumosa* as a source of bioactive natural products, particularly through bioassay-guided fractionation, isolation and structural confirmation of active constituents, evaluation of mechanisms of action, assessment of cytotoxic selectivity toward non-malignant cells, and subsequent in vivo studies. Such investigations will be necessary to determine whether the observed in vitro activities can be translated into meaningful biomedical or veterinary applications.

## 5. Conclusions

The present study provides evidence that *Stipagrostis plumosa* is a chemically diverse desert grass with promising biological potential. The complementary activities observed among its solvent fractions indicate that solvent-dependent enrichment of different metabolite groups may contribute to distinct biological effects. In particular, the differential antioxidant, antimicrobial, and cytotoxic responses observed among the fractions support the value of solvent fractionation as an approach for identifying biologically relevant constituents of this underexplored species.

The chemical profiles obtained by GC–MS further support the presence of diverse metabolite classes that may contribute collectively to the observed activities. However, the biological effects cannot be assigned to individual compounds solely on the basis of tentative GC–MS identification, and potential additive or synergistic interactions among constituents should be considered. Importantly, the antimicrobial activity observed against both reference microorganisms and animal-derived isolates highlights the potential relevance of *S. plumosa* for the exploration of plant-derived antimicrobial candidates, particularly in veterinary contexts where alternative approaches to antimicrobial resistance are needed.

The present findings should nevertheless be regarded as an initial indication of biological potential rather than evidence of therapeutic efficacy. Further research should focus on bioassay-guided fractionation and isolation of the active constituents, followed by definitive structural characterization using authentic standards and complementary spectroscopic techniques. Mechanistic studies, evaluation of selectivity against non-malignant cells, comprehensive toxicity assessment, and in vivo validation are also required. Investigation of the most active fractions against multidrug-resistant veterinary pathogens may further clarify their practical relevance and help assess the feasibility of developing *S. plumosa*-derived compounds or standardized fractions for pharmaceutical and veterinary applications.

Overall, this study establishes a scientific basis for further investigation of *S. plumosa* as a potential source of natural bioactive products and contributes to the broader exploration of underutilized plants adapted to arid environments.

## Figures and Tables

**Table 1:** Total phenolic content (TPC) and total flavonoid content (TFC) of the crude methanol extract of *Stipagrostis plumosa*.

Plant Species	Total Phenolic Content (mg GAE/g Dry Extract)	Total Flavonoid Content (mg QE/g Dry Extract)
*Stipagrostis plumosa*	72.1 ± 0.01	13.6 ± 0.01

**Table 2:** Major bioactive compounds identified by GC–MS in the ethyl acetate and chloroform fractions of *Stipagrostis plumosa*.

No.	Compound	RT (min)	Ethyl Acetate (% Area)	Chloroform (% Area)	Quality (%)
1	Stigmasta-3,5-dien-7-one	29.253	14.70	−	90
2	Vanillic acid	15.687	7.18	−	95
3	2-Pentadecanone, 6,10,14-trimethyl	19.234	5.83	−	91
4	Hexadecanoic acid, methyl ester	20.287	−	5.28	97
5	2-Methoxy-4-vinylphenol	11.504	4.77	−	90
6	Dodecanoic acid	15.452/15.469	4.77	2.77	98
7	6-Hydroxy-4,4,7a-trimethyl-5,6,7,7a-tetrahydrobenzofuran-2(4H)-one	18.576	−	4.67	96
8	Tetradecanoic acid	18.244/18.256	4.39	4.30	99
9	Octadecanoic acid	23.125	−	3.71	99
10	4,8,12,16-Tetramethylheptadecan-4-olide	25.111	2.88	−	98
11	Vanillin	13.003	2.51	−	96
12	α-Tocospiro A	29.740	−	1.31	83
13	2,4-Di-tert-butylphenol	14.617	−	1.01	96
Cumulative peak area of the major compounds (%)			47.03	23.05	

**Table 3:** IC_50_ values of the crude methanol extract and solvent fractions of *Stipagrostis plumosa* against A549 and MCF-7 cancer cell lines.

Cell Lines	Crude Methanol	Hexane	Chloroform	Ethyl Acetate	*n*-Butanol	Doxorubicin
A549	ND	238.94 ± 4.9	194.17 ± 2.5	257.52 ± 3.8	261.08 ± 1.7	3.52 ± 0.05
MCF-7	ND	304.91± 1.3	173.52 ± 2.7	386.36 ± 3.2	328.37 ± 2.5	2.5 ± 0.03

Data are presented as mean ± SD (*n* = 3). ND: No detectable cytotoxic activity under the tested concentrations. IC_50_: concentration required to inhibit 50% of cell viability.

**Table 4:** Inhibition zone diameters (mm) of the crude methanol extract and solvent fractions of *Stipagrostis plumosa* against reference bacterial and fungal strains.

Extract	Gram-Positive Bacteria		Gram-Negative Bacteria		Fungi	
	*S. aureus* ATCC 29213	*E. faecalis* ATCC 29212	*E. coli* ATCC 25922	*S. typhimurium* ATCC 14028	*C. albicans* ATCC 60193	*C. tropicalis* ATCC 66029
*n*-Butanol	11.17 ± 0.21	10.46 ± 0.32	11.13 ± 0.15	9.27 ± 0.25	10.23 ± 0.32	10.17 ± 0.21
Ethyl acetate	15.27 ± 0.31	12.30 ± 0.26	19.30 ± 0.30	16.20 ± 0.10	14.70 ± 0.20	13.40 ± 0.26
Chloroform	14.20 ± 0.26	11.23 ± 0.15	12.23 ± 0.25	13.50 ± 0.20	11.33 ± 0.29	11.20 ± 0.26
*n*-Hexane	13.10 ± 0.10	9.40 ± 0.10	10.63 ± 0.15	11.30 ± 0.30	9.26 ± 0.31	9.23 ± 0.25
Crude methanol	14.56 ± 0.25	11.30 ± 0.20	16.20 ± 0.26	14.10 ± 0.10	13.36 ± 0.32	12.40 ± 0.20
Ether	12.22 ± 0.29	10.28 ± 0.26	10.64 ± 0.13	9.15 ± 0.17	ND	ND
Acetone	8.25 ± 0.27	11.40 ± 0.26	9.26 ± 0.25	10.12 ± 0.13	9.34 ± 0.14	8.22 ± 0.29
Hot aqueous	6.20 ± 0.22	7.43 ± 0.31	8.49 ± 0.25	8.36 ± 0.23	ND	ND
Cold aqueous	9.21 ± 0.10	8.56 ± 0.14	9.20 ± 0.20	10.23 ± 0.11	7.55 ± 0.13	9.17 ± 0.15
Negative control (Distilled water)	ND	ND	ND	ND	ND	ND
Positive control (Ciprofloxacin)	21.50 ± 0.50	18.30 ± 0.20	19.83 ± 0.76	18.37 ± 0.35	NT	NT
Positive control (Nystatin)	NT	NT	NT	NT	18.17 ± 0.15	19.87 ± 0.15

Data are presented as mean ± SD (*n* = 3). ND: No detectable inhibition zone. NT: Not tested.

**Table 5:** Inhibition zone diameters (mm) of the crude methanol extract and solvent fractions of *Stipagrostis plumosa* against animal-derived bacterial and fungal isolates.

Microorganism	HA	CA	Hex	But	EtOAC	E	A	CHCL3	CM	C-VE (DW)	C + VE (Cipro)	C + VE (Ny)
Gram-negative bacteria												
*Pseudomonas aeruginosa*	9.28 ± 0.26	10.37 ± 0.25	10.26 ± 0.18	8.17 ± 0.21	15.27 ± 0.31	11.30 ± 0.20	10.13 ± 0.14	12.63 ± 0.15	13.20 ± 0.26	ND	31.23 ± 0.25	NT
*Escherichia coli*	8.10 ± 0.10	11.30 ± 0.26	15.40 ± 0.30	10.43 ± 0.15	20.20 ± 0.11	14.24 ± 0.25	12.14 ± 0.17	16.17 ± 0.15	17.73 ± 0.21	ND	19.23 ± 0.21	NT
*Salmonella typhimurium*	9.12 ± 0.13	10.50 ± 0.10	13.19 ± 0.24	12.22 ± 0.11	17.30 ± 0.30	10.20 ± 0.26	11.33 ± 0.21	14.25 ± 0.22	15.22 ± 0.29	ND	30.13 ± 0.32	NT
*Klebsiella pneumoniae*	7.22 ± 0.13	8.18 ± 0.23	10.17 ± 0.19	7.25 ± 0.14	12.12 ± 0.11	9.21 ± 0.27	8.25 ± 0.17	11.46 ± 0.25	11.53 ± 0.15	ND	34.20 ± 0.20	NT
Gram-positive bacteria												
*Bacillus cereus*	9.53 ± 0.21	11.43 ± 0.25	13.40 ± 0.36	12.25 ± 0.13	19.19 ± 0.16	11.36 ± 0.15	10.24 ± 0.13	14.24 ± 0.25	18.27 ± 0.31	ND	34.10 ± 0.20	NT
*Streptococcus pyogenes*	8.38 ± 0.16	9.32 ± 0.20	11.43 ± 0.20	10.34 ± 0.10	15.19 ± 0.18	10.30 ± 0.20	9.28 ± 0.26	12.30 ± 0.10	13.47 ± 0.25	ND	R	NT
*Staphylococcus aureus*	7.39 ± 0.10	10.44 ± 0.15	12.39 ± 0.10	11.50 ± 0.26	17.40 ± 0.30	10.33 ± 0.25	11.43 ± 0.38	14.42 ± 0.18	15.34 ± 0.11	ND	25.37 ± 0.40	NT
*Enterococcus faecium*	7.10 ± 0.10	8.10 ± 0.11	11.43 ± 0.06	10.13 ± 0.15	14.10 ± 0.10	9.60 ± 0.10	10.28 ± 0.16	12.60 ± 0.30	13.30 ± 0.26	ND	35.30 ± 0.30	NT
Fungi												
*Candida albicans*	ND	7.49 ± 0.12	10.42 ± 0.19	7.42 ± 0.10	16.53 ± 0.25	9.31 ± 0.30	8.38 ± 0.15	11.46 ± 0.32	14.35 ± 0.30	ND	NT	16.33 ± 0.31

Butanol is denoted as But, ethyl acetate is represented as EtOAC, chloroform is indicated by CHCL3, and n-hexane is referred to as Hex. The crude methanol extract is abbreviated as CM, while acetone is labeled as A, and ether is represented as E. The cold aqueous extract is denoted as CA, and the hot aqueous extract is indicated as HA. Control positive is abbreviated as C + VE, and control negative is denoted as C-VE. Ciprofloxacin is represented as Cipro (5 µg), and nystatin is indicated as Ny (30 µg). Data are presented as mean ± SD (*n* = 3). ND: No detectable inhibition zone. NT: Not tested. DW: Distilled water. R: Resistant.

**Table 6:** Minimum inhibitory concentration (MIC) and minimum bactericidal/fungicidal concentration (MBC/MFC) of the three most active *Stipagrostis plumosa* extracts against the tested microorganisms.

Microorganism	Ethyl Acetate MIC (mg/mL)	Ethyl Acetate MBC/MFC (mg/mL)	Crude Methanol MIC (mg/mL)	Crude Methanol MBC/MFC (mg/mL)	Chloroform MIC (mg/mL)	Chloroform MBC/MFC (mg/mL)
*Pseudomonas aeruginosa*	25	50	50	75	50	75
*Escherichia coli*	5	25	5	25	25	50
*Enterococcus faecium*	50	75	50	75	50	75
*Salmonella typhimurium*	25	50	25	50	50	75
*Klebsiella pneumoniae*	50	75	50	75	50	75
*Bacillus cereus*	5	25	5	25	50	75
*Streptococcus pyogenes*	25	50	50	75	50	75
*Staphylococcus aureus*	5	25	25	50	50	75
*Candida albicans*	25	50	50	75	50	75
*Staphylococcus aureus* ATCC 29213	25	50	50	75	50	75
*Enterococcus faecalis* ATCC 29212	50	75	50	75	50	75
*Escherichia coli* ATCC 25922	5	25	25	50	50	75
*Salmonella typhimurium* ATCC 14028	25	50	50	75	50	75
*Candida albicans* ATCC 60193	50	75	50	75	50	75
*Candida tropicalis* ATCC 66029	50	75	50	75	50	75

Values represent the minimum inhibitory concentration (MIC) and minimum bactericidal/fungicidal concentration (MBC/MFC) of the three most active solvent extracts. MIC was defined as the lowest concentration that completely inhibited visible microbial growth, whereas MBC/MFC was defined as the lowest concentration that produced no visible colony growth after subculture on agar plates. MIC and MBC/MFC determinations were performed in three independent experiments, and identical endpoint concentrations were obtained across all three replicates for each microorganism. Accordingly, no inter-replicate variation was observed (SD = 0.00), and the results are presented as single endpoint concentrations.

## Data Availability

The original contributions presented in this study are included in the article/Supplementary Materials. Further inquiries can be directed to the corresponding authors.

## Supplementary Materials

The following supporting information can be downloaded at: https://www.mdpi.com/article/10.3390/biology15161428/s1, Figure S1: Complete GC–MS chromatogram of the ethyl acetate extract of *Stipagrostis plumosa*; Figure S2. Complete GC–MS chromatogram of the chloroform extract of *Stipagrostis plumosa*; Table S1: Complete GC–MS library-search output of the ethyl acetate extract of *Stipagrostis plumosa*; Table S2. Complete GC–MS library-search output of the chloroform extract of *Stipagrostis plumosa*.
