# Supplementary material for: Phytochemical Profiling, Antioxidant, Antimicrobial and Cytotoxic Activities of Stipagrostis plumosa (Poaceae): Potential Implications for Human and Veterinary Health
Source: Biology (Basel). 2026 Aug 19;15(16):1428. doi: 10.3390/biology15161428 (PMC13510140; doi:10.3390/biology15161428)

Supplementary S1:

Table S1. Complete GC–MS library-search output of the ethyl acetate extract of *Stipagrostis plumosa*.

No.	RT (min)	Peak area (Ab*s)	Tentative library hit	Library match quality (%)	Relative peak area (%)	MW (amu)	CAS No.
1	3.63	290916	Cyclopentanol, 3-methyl-	64	0.21	100.089	018729-48-1
2	4.706	454064	1,5-Heptadiene-3,4-diol, 2,5-dimethyl-	40	0.33	156.115	022607-16-5
3	5.416	1318809	Cyclopropane, 1,1,2,2-tetramethyl-	72	0.95	98.11	004127-47-3
4	7.596	3305841	Undecane	93	2.39	156.188	001120-21-4
5	9.404	575032	Eicosane	58	0.42	282.329	000112-95-8
6	9.97	6046169	Benzofuran, 2,3-dihydro-	74	4.37	120.058	000496-16-2
8	11.504	6603111	2-Methoxy-4-vinylphenol	90	4.77	150.068	007786-61-0
9	12.133	552772	Phenol, 2,6-dimethoxy-	96	0.40	154.063	000091-10-1
10	12.219	674509	3-Heptyne	52	0.49	96.094	002586-89-2
11	12.7	456964	Benzaldehyde, 4-hydroxy-	93	0.33	122.037	000123-08-0
12	13.003	3479482	Vanillin	96	2.51	152.047	000121-33-5
13	14.01	491893	Acetophenone, 4'-hydroxy-	45	0.36	136.052	000099-93-4
14	14.37	744437	Apocynin	68	0.54	166.063	000498-02-2
15	14.531	2840051	Butylated Hydroxytoluene	98	2.05	220.183	000128-37-0
16	15.452	6604965	Dodecanoic acid	98	4.77	200.178	000143-07-7
17	15.687	9945886	Vanillic acid	95	7.18	168.042	000121-34-6
18	16.9	2722731	Benzaldehyde, 4-hydroxy-3,5-dimethoxy-	93	1.97	182.058	000134-96-3
19	17.306	1334701	1,4-Hexadiene, 2,3,4,5-tetramethyl-	64	0.96	138.141	051504-54-2
20	17.678	1205950	Methyl trans-9-(2-butylcyclopentyl)nonanoate	45	0.87	296.272	108708-61-8
21	17.735	1068987	2,5-DMMA	59	0.77	209.142	054687-43-3

22	17.809	1086500	Methoxyphenamine	58	0.78	179.131	000093-30-1
23	18.067	4054311	4-((1E)-3-Hydroxy-1-propenyl)-2-methoxyphenol	97	2.93	180.079	1000297-95-5
24	18.244	6086163	Tetradecanoic acid	99	4.39	228.209	000544-63-8
25	18.565	5353424	6-Hydroxy-4,4,7a-trimethyl-5,6,7,7a-tetrahydrobenzofuran-2(4H)-one	98	3.87	196.11	073410-02-3
26	18.719	2103931	1,3-Benzenediol, 2-methyl-	55	1.52	124.052	000608-25-3
27	19.234	8077811	2-Pentadecanone, 6,10,14-trimethyl-	91	5.83	268.277	000502-69-2
28	19.812	659306	Citral	43	0.48	152.12	005392-40-5
29	20.224	475264	Cyclododecanone, 2-methylene-	86	0.34	194.167	003045-76-9
30	20.281	6587735	Pentadecanoic acid, 14-methyl-, methyl ester	98	4.76	270.256	005129-60-2
32	21.5	924034	Methyl 9-methyltetradecanoate	50	0.67	256.24	213617-69-7
33	22.49	1366459	Bromoacetic acid, octadecyl ester	42	0.99	390.213	018992-03-5
34	22.604	1957254	3,4-Methylenedioxy-N-benzylcathinone	53	1.41	283.121	1000386-14-3
35	22.662	2753633	Methyl stearate	97	1.99	298.287	000112-61-8
36	22.856	1155059	Palmitoleic acid	43	0.83	254.225	000373-49-9
37	23.114	4158215	Octadecanoic acid	99	3.00	284.272	000057-11-4
38	23.32	860888	Hexadecanoic acid, 1,1-dimethylethyl ester	83	0.62	312.303	031158-91-5
39	23.405	1086865	Octadecanoic acid, ethyl ester	96	0.78	312.303	000111-61-5
40	24.161	3135312	Triacontyl trifluoroacetate	50	2.26	534.462	1000351-75-7
41	25.111	3987671	4,8,12,16-Tetramethylheptadecan-4-olide	98	2.88	324.303	096168-15-9
42	25.483	673076	Hexanedioic acid, bis(2-ethylhexyl) ester	53	0.49	370.308	000103-23-1
43	26.919	2295174	Diethyl Phthalate	50	1.66	222.089	000084-66-2

44	28.984	558596	2-Ethylacridine	60	0.40	207.105	055751-83-2
45	29.253	20356295	Stigmasta-3,5-dien-7-one	90	14.70	410.355	002034-72-2
46	29.665	637057	2-Ethylacridine	41	0.46	207.105	055751-83-2
47	29.734	2284981	Methyl 2-[1-(4-methylphenyl)hydrazino]propanoate	48	1.65	208.121	1000305-34-3
48	30.432	715388	2'-Hydroxypropiophenone, TMS derivative	46	0.52	222.108	033342-87-9
49	31.033	638595	Thymol, TMS derivative	38	0.46	222.144	055012-80-1
50	31.21	2394987	2-(Acetoxymethyl)-3-(methoxycarbonyl)biphenylene	46	1.73	282.089	093103-70-9
51	31.616	1154902	1,2-Benzenediol, 3,5-bis(1,1-dimethylethyl)-	46	0.83	222.162	001020-31-1
52	31.685	193919	2-tert-Butylphenol, tert-butyldimethylsilyl ether	43	0.14	264.191	860465-21-0
		138490075			100.00		

Peak numbers correspond to the original GC–MS library-search output; therefore, numbering gaps may occur where no library-assigned hit was reported.

Figure S1. Complete GC–MS chromatogram of the ethyl acetate extract of *Stipagrostis plumosa*.

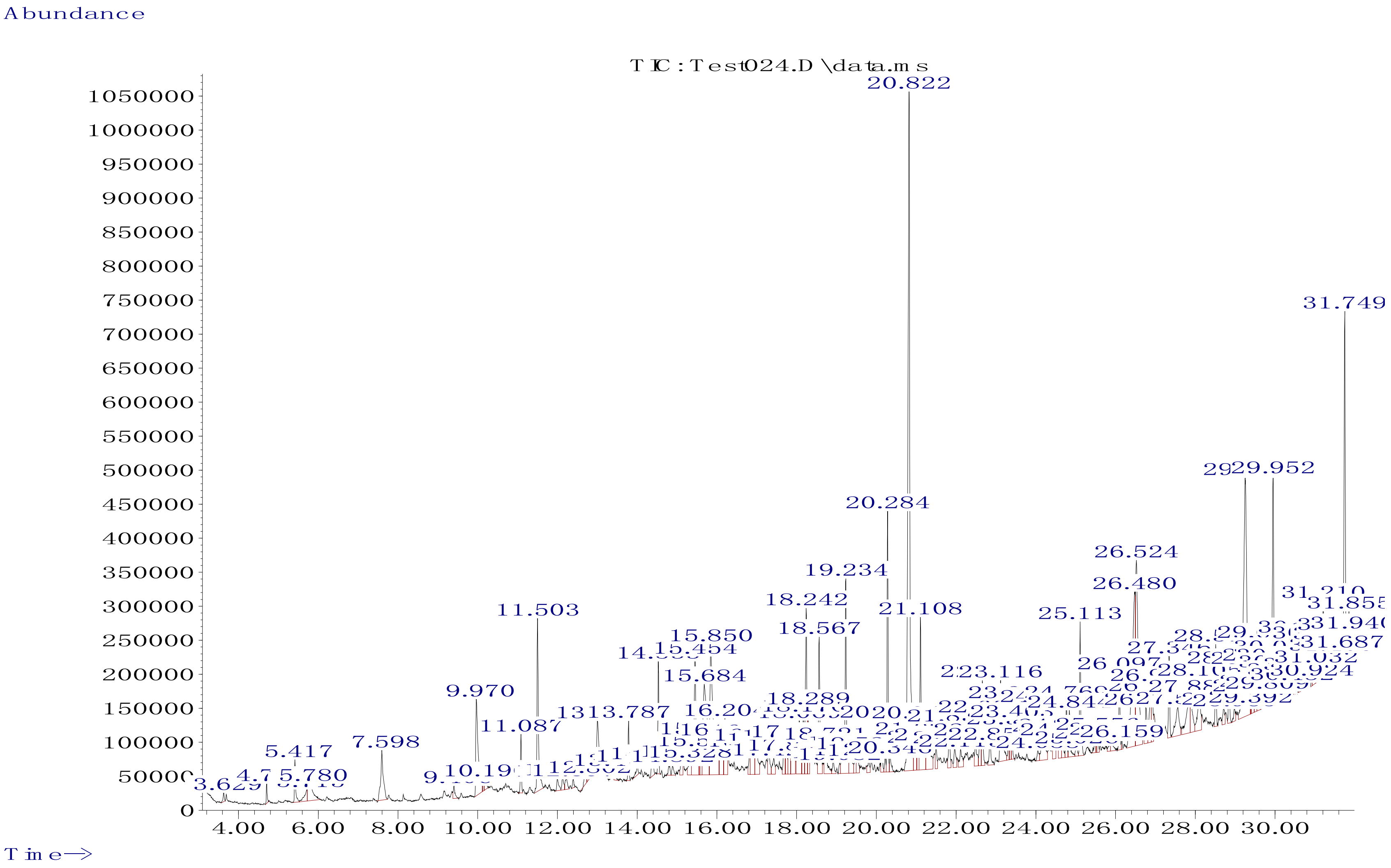

Supplement: Supplementary file 1 [file biology-15-01428-s001.zip › S1.pdf]
