# Supplementary material for: Phytochemical Profiling, Antioxidant, Antimicrobial and Cytotoxic Activities of Stipagrostis plumosa (Poaceae): Potential Implications for Human and Veterinary Health
Source: Biology (Basel). 2026 Aug 19;15(16):1428. doi: 10.3390/biology15161428 (PMC13510140; doi:10.3390/biology15161428)

Supplementary S2:

Table S2. Complete GC–MS library-search output of the chloroform extract of *Stipagrostis plumosa*.

No.	RT (min)	Peak area (Ab*s)	Tentative library hit	Library match quality (%)	Relative peak area (%)	MW (amu)	CAS No.
1	3.647	351277	Cyclopentanol, 3-methyl-	64	0.17	100.089	018729-48-1
2	4.723	415182	2-(2-Ethoxyethoxy)ethyl pentanoate	38	0.20	218.152	1000367-01-6
3	5.433	1116412	2,6-Octadiene, 2,4-dimethyl-	72	0.53	138.141	063843-03-8
4	5.833	527321	Benzene, 1-ethyl-3-methyl-	50	0.25	120.094	000620-14-4
5	6.228	387590	Benzyl chloride	76	0.19	126.024	000100-44-7
6	7.601	2463488	Undecane	87	1.18	156.188	001120-21-4
7	9.41	931380	Hexadecane	74	0.44	226.266	000544-76-3
8	10.136	368195	D-Campholic acid	38	0.18	170.131	031147-56-5
9	11.509	2715179	2-Methoxy-4-vinylphenol	96	1.30	150.068	007786-61-0
10	12.007	650885	2,3,4,5-Tetrahydropyridazine	38	0.31	84.069	000694-06-4
11	12.242	511031	3-Heptyne	49	0.24	96.094	002586-89-2
12	12.408	530021	Palmitoleic acid	30	0.25	254.225	000373-49-9
13	13.032	1639049	Vanillin	90	0.78	152.047	000121-33-5
14	13.449	897048	Methyl 8-oxooctanoate	38	0.43	172.11	004316-48-7
15	14.113	548953	Oxalic acid, hexyl tetradecyl ester	22	0.26	370.308	1000309-24-8
16	14.376	617759	Apocynin	38	0.30	166.063	000498-02-2
17	14.411	356978	N-Methylsalicylamide	46	0.17	151.063	001862-88-0
18	14.531	3986316	Butylated Hydroxytoluene	99	1.90	220.183	000128-37-0
19	14.617	2119331	2,4-Di-tert-butylphenol	96	1.01	206.167	000096-76-4

20	14.8	2770832	Propylamine, 1,1,2,2-tetramethyl-, hydrochloride	53	1.32	115.136	029772-54-1
21	14.897	2606901	Tapentadol, trimethylsilyl ether	59	1.25	293.217	1000417-23-2
22	15.017	2204442	1-Propanamine, 2-chloro-N,N-dimethyl-	64	1.05	121.066	000108-14-5
23	15.12	3337011	Methoxyphenamine	47	1.59	179.131	000093-30-1
24	15.469	5805198	Dodecanoic acid	98	2.77	200.178	000143-07-7
25	15.555	564143	2,5-DMMA	35	0.27	209.142	054687-43-3
26	15.612	1489545	2-Thiophenecarboxaldehyde, oxime	46	0.71	127.009	029683-84-9
27	15.692	4320122	Vanillic acid	95	2.06	168.042	000121-34-6
28	15.784	729224	Dehydroacetic Acid	46	0.35	168.042	000520-45-6
29	15.864	10928895	Benzoic acid, 4-hydroxy-3-methoxy-, ethyl ester	47	5.22	196.074	000617-05-0
30	16.099	1768899	Ethanethioamide, N-phenyl-	38	0.85	151.046	000637-53-6
31	16.207	1833825	N-(Trifluoroacetyl)-N,O,O',O''-tetrakis(trimethylsilyl)norepinephrine	43	0.88	553.214	1000072-26-7
32	16.9	2025592	Benzaldehyde, 4-hydroxy-3,5-dimethoxy-	95	0.97	182.058	000134-96-3
33	17.243	360513	4-Chlorobutyric acid, pentadecyl ester	49	0.17	332.248	1000340-23-4
34	17.306	1131324	2-(1,4,4-Trimethyl-cyclohex-2-enyl)-ethanol	70	0.54	168.151	1000190-92-3
35	17.58	1611264	O-Desmethyl-cis-tramadol	59	0.77	249.173	144830-14-8
36	17.609	1796819	Stearyltrimethylammonium chloride	59	0.86	347.332	000112-03-8
37	18.176	4559395	9-Borabicyclo[3.3.1]nonane, 9-(2-propen-1-yloxy)-	42	2.18	178.153	1000162-57-7
38	18.256	8996376	Tetradecanoic acid	99	4.30	228.209	000544-63-8
39	18.576	9773951	6-Hydroxy-4,4,7a-trimethyl-5,6,7,7a-tetrahydrobenzofuran-2(4H)-one	96	4.67	196.11	073410-02-3

40	18.725	4180419	2-Methylresorcinol, acetate	68	2.00	166.063	1000352-78-7
41	18.919	1156100	Carbonic acid, eicosyl vinyl ester	49	0.55	368.329	1000382-54-3
42	19.137	1669252	Methyl 6,8-dodecadienyl ether	53	0.80	196.183	1000130-99-9
43	19.171	1023307	Benzoic acid, 4-hydroxy-3-methoxy-, methyl ester	87	0.49	182.058	003943-74-6
44	19.234	6043129	2-Pentadecanone, 6,10,14-trimethyl-	90	2.89	268.277	000502-69-2
45	19.383	2278828	Apocynin	38	1.09	166.063	000498-02-2
46	19.532	2002139	Phthalic acid, cyclohexylmethyl isohexyl ester	47	0.96	346.214	1000309-07-1
47	19.955	221992	Cyclotridecane	50	0.11	182.203	000295-02-3
48	20.092	386556	Docosyl heptyl ether	45	0.18	424.464	1000406-39-7
49	20.23	753452	Cycloheptanecarboxylic acid	42	0.36	142.099	001460-16-8
50	20.287	11050664	Hexadecanoic acid, methyl ester	97	5.28	270.256	000112-39-0
51	20.344	541636	Cyclododecane	44	0.26	168.188	000294-62-2
52	20.728	688875	Dibutyl phthalate	64	0.33	278.152	000084-74-2
54	21.283	1573175	Estra-1,3,5(10)-trien-17.beta.-ol	62	0.75	256.183	002529-64-8
55	21.5	1489422	Heptadecanoic acid, methyl ester	91	0.71	284.272	001731-92-6
56	21.975	1480465	Heptadecanoic acid	45	0.71	270.256	000506-12-7
57	22.215	2329031	Trifluoroacetic acid, pentadecyl ester	92	1.11	324.228	959010-23-2
58	22.496	1168605	cis-Vaccenic acid	38	0.56	282.256	000506-17-2
59	22.593	4649745	1-(4-Methylphenyl)piperazine	80	2.22	176.131	039593-08-3
60	22.662	5251213	Methyl stearate	98	2.51	298.287	000112-61-8

61	22.862	1698394	Docosyl heptyl ether	45	0.81	424.464	1000406-39-7
62	23.125	7772641	Octadecanoic acid	99	3.71	284.272	000057-11-4
63	23.325	5767813	Hexadecanoic acid, butyl ester	96	2.76	312.303	000111-06-8
64	23.571	1166740	Cyclododecanone, 2-methylene-	87	0.56	194.167	003045-76-9
65	23.777	627086	Oleic Acid	45	0.30	282.256	000112-80-1
66	24.046	537282	Cyclododecanone, 2-methylene-	80	0.26	194.167	003045-76-9
67	24.166	2112876	Heptacosyl trifluoroacetate	45	1.01	492.415	1000351-75-8
68	24.247	788346	Tetrapentacontane, 1,54-dibromo-	55	0.38	914.682	1000156-09-4
69	24.407	690632	Dotriacontyl pentafluoropropionate	49	0.33	612.49	1000351-81-4
70	24.464	1010692	Cyclopentadecane	70	0.48	210.235	000295-48-7
71	24.521	633246	Carbonic acid, decyl hexadecyl ester	76	0.30	426.407	1000383-16-5
72	24.613	1612729	E-8-Methyl-9-tetradecen-1-ol acetate	62	0.77	268.24	1000130-81-4
73	24.842	4876814	2-Methyl-1,2,4-triazolo(2,3-a)pyrazine	47	2.33	134.059	020914-69-6
74	25.025	1266483	1-Docosene	64	0.61	308.344	001599-67-3
75	25.116	6636421	4,8,12,16-Tetramethylheptadecan-4-olide	99	3.17	324.303	096168-15-9
76	25.162	1415039	1-Hexadecanesulfonyl chloride	60	0.68	324.189	038775-38-1
77	25.442	838853	Oleic Acid	50	0.40	282.256	000112-80-1
78	25.483	1737524	Diisooctyl adipate	87	0.83	370.308	001330-86-5
79	25.895	1015804	Tetrapentacontane, 1,54-dibromo-	70	0.49	914.682	1000156-09-4

80	26.163	466855	Octacosyl heptafluorobutyrate	90	0.22	606.425	1000351-83-6
81	26.524	642809	Pentadecafluorooctanoic acid, octadecyl ester	64	0.31	666.255	1000406-04-8
82	26.919	3351482	Bis(2-ethylhexyl) phthalate	74	1.60	390.277	000117-81-7
83	27.256	883052	1-Eicosene	53	0.42	280.313	003452-07-1
84	28.172	264640	Pyridine-3-carboxamide, oxime, N-(2-trifluoromethylphenyl)-	90	0.13	281.078	288246-53-7
85	28.515	3575422	Tetracosamethyl-cyclododecasiloxane	80	1.71	888.226	018919-94-3
86	28.979	354623	Methyl 2-[1-(4-methylphenyl)hydrazino]propanoate	60	0.17	208.121	1000305-34-3
87	29.665	1007324	Acridine-9-carbaldehyde	50	0.48	207.068	1000318-45-4
88	29.74	2739856	.alpha.-Tocospiro A	83	1.31	462.371	601490-40-8
89	29.808	264601	2-Ethylacridine	46	0.13	207.105	055751-83-2
90	30.987	1767937	2-Ethylacridine	38	0.84	207.105	055751-83-2
91	31.21	2848896	2-(Acetoxymethyl)-3-(methoxycarbonyl)biphenylene	41	1.36	282.089	093103-70-9
92	31.393	1067345	1,2-Benzenediol, 3,5-bis(1,1-dimethylethyl)-	46	0.51	222.162	001020-31-1
93	31.456	2747667	Acridine-9-carbaldehyde	43	1.31	207.068	1000318-45-4
94	31.857	4062222	Anthracene, 9,10-diethyl-9,10-dihydro-	49	1.94	236.157	046868-29-5
95	31.92	1377713	2-tert-Butylphenol, tert-butyldimethylsilyl ether	45	0.66	264.191	860465-21-0
		209313530			100.00		

Peak numbers correspond to the original GC–MS library-search output; therefore, numbering gaps may occur where no library-assigned hit was reported.

Figure S2. Complete GC–MS chromatogram of the chloroform extract of *Stipagrostis plumosa*.

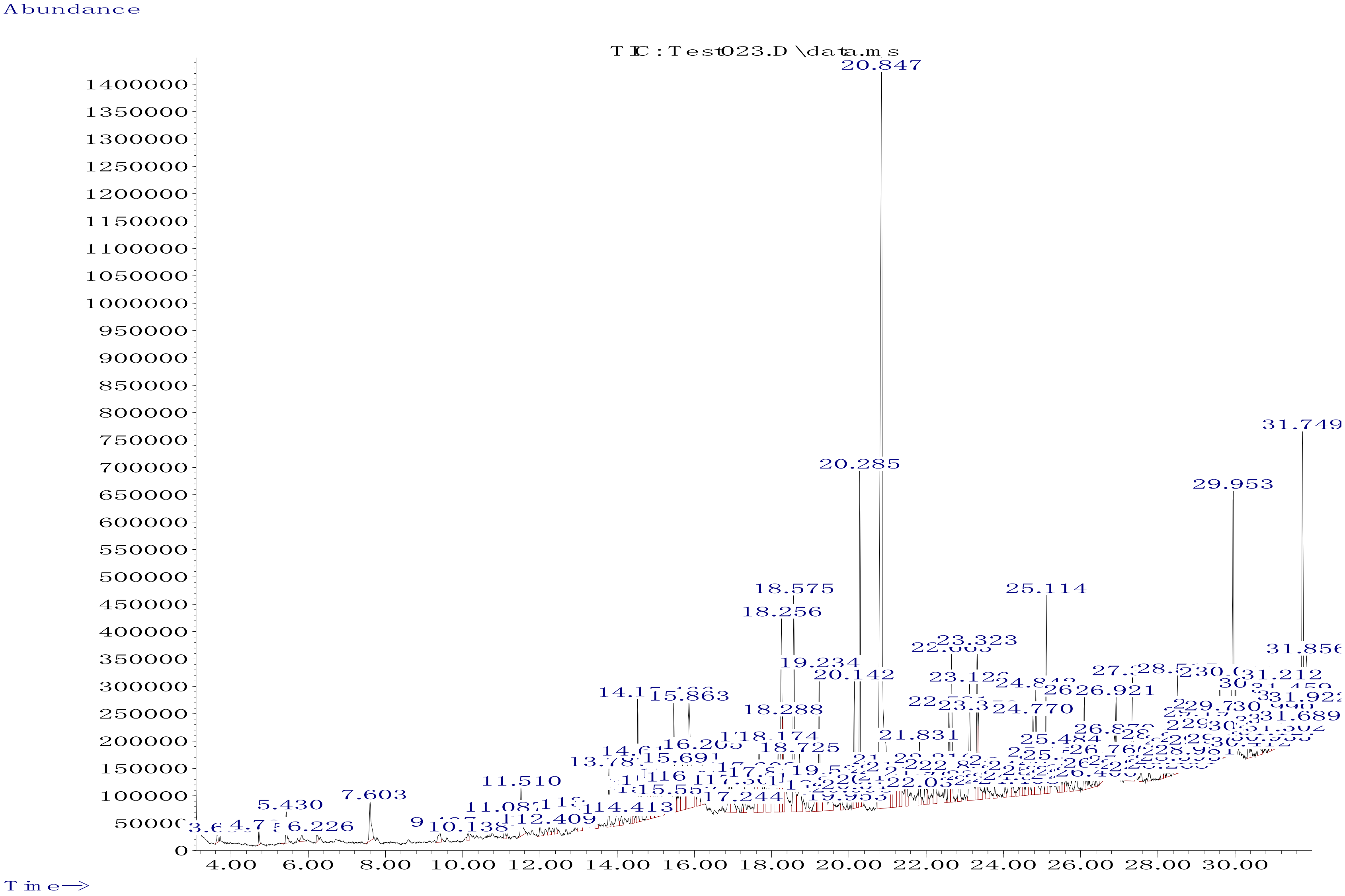

Supplement: Supplementary file 1 [file biology-15-01428-s001.zip › S2.pdf]
